# Managing health in dual diagnosis: narratives from individuals with schizophrenia and diabetes

**DOI:** 10.3389/fpsyt.2025.1713082

**Published:** 2026-01-12

**Authors:** Matilde Zerne Nilsson, Carina Sparud-Lundin, Katarina Eeg-Olofsson, Peter Sand, Christina Hagberg, Christopher Holmberg

**Affiliations:** 1 Institute of Health and Care Sciences, University of Gothenburg, Gothenburg, Sweden; 2 Department of Medicine, Sahlgrenska University Hospital, Gothenburg, Sweden; 3 Department of Molecular and Clinical Medicine, Institute of Medicine, University of Gothenburg, Gothenburg, Sweden; 4 Centre of Registers, Västra Götalandsregionen, Gothenburg, Sweden; 5 Department of Psychology, University of Gothenburg, Gothenburg, Sweden; 6 Department of Psychotic disorders, Sahlgrenska University Hospital, Gothenburg, Sweden

**Keywords:** cognitive dysfuntcion, diabetes mellitus, health management, lifestyle, schizophrenia

## Abstract

**Introduction:**

Schizophrenia, often accompanied by cognitive impairments, increases the risk of diabetes, complicating self-care and overall health management. Exploring the lived experiences of individuals who face both mental and physical illness—and, in many cases, pronounced cognitive or functional challenges—is critical for developing more effective and integrated care strategies.

**Aim:**

This study examined the daily lives of individuals living with schizophrenia and diabetes, focusing on their health-related experiences and interactions with healthcare services.

**Methods:**

Twenty-two participants diagnosed with schizophrenia and type 1 or type 2 diabetes, including individuals with significant cognitive and functional impairments, were interviewed in a qualitative study. Data were analyzed using thematic analysis grounded in descriptive phenomenology.

**Results:**

Three themes captured participants’ experiences: (1) *Perceptions of health shaped by personal narratives*, including the role of insight, autonomy, and meaning; (2) *Struggles with navigating health in daily life*, as participants balanced medication effects, symptoms, and practical constraints while attempting recommended routines; and (3) *Need for formal support in managing health*, with valued help often being concrete, everyday assistance alongside respectful, encouraging relationships with providers.

**Conclusion:**

These findings highlight the importance of holistic, coordinated care that integrates mental and physical health, addresses practical barriers, and fosters self-understanding. By including participants with cognitive impairments, this study adds new insight into how such challenges influence daily health management and the types of support needed to sustain engagement in care.

## 1 Introduction

Schizophrenia is a severe, often lifelong mental illness characterized by a misperception of reality, frequently accompanied by delusions, hallucinations, and disorganized thinking (1, 2). Cognitive impairment, increasingly recognized as a core feature of schizophrenia, affects approximately 80% of individuals with the disorder to varying degrees in the neurocognitive and social cognitive domains (3–5). Such cognitive deficits are also more pronounced in schizophrenia than in other mental illnesses such as bipolar disorder and depression (6).

Individuals with schizophrenia are at a substantially higher risk of developing metabolic disorders, including diabetes mellitus (7, 8). Studies have indicated that the prevalence of diabetes in the population with schizophrenia is 2–5 times greater than in the general population (9–11). The increased risk of type 2 diabetes in schizophrenia is mainly explained by the metabolic side effects of antipsychotic medications and lifestyle factors, while schizophrenia itself, gender, and disparities in healthcare also contribute to this elevated risk (12–18). Metabolic dysregulation not only contributes to reducing life expectancy by 15–20 years and increases mortality rates among individuals with schizophrenia but also exacerbates cognitive impairments (19). This link may involve insulin resistance, cerebrovascular changes, and low-grade inflammation, all of which are associated with cognitive impairments in schizophrenia, including deficits in attention, memory, and executive function (20, 21). Cognitive impairments in schizophrenia significantly interfere with real-life functioning, often more than positive or negative symptoms and can hinder an individual’s ability to manage daily activities, including self-care practices that are essential for maintaining physical health (20, 22).

Clinical guidelines advocate for integrating physical and mental healthcare to reduce the mortality gap and improve quality of life (23, 24). Meta-analyses of randomized controlled trials have demonstrated the effectiveness of both pharmacological and non-pharmacological interventions for improving physical health outcomes in schizophrenia. Individual lifestyle counselling and exercise had the strongest effects on weight reduction, with additional benefits seen from psychoeducation and certain pharmacological strategies (e.g., aripiprazole augmentation, topiramate, metformin). These results indicate that structured interventions can improve physical health, but they provide little insight into how individuals with both schizophrenia and diabetes experience or implement such changes in their daily lives (25).

In clinical practice, implementing these recommended lifestyle and self-management interventions often proves challenging. Factors such as cognitive impairments, negative symptoms, and socioeconomic disadvantage can make it difficult for individuals to initiate and sustain health-promoting behaviors without coordinated professional and social support (26).

Previous research has highlighted how such challenges manifest in everyday life, showing that people with serious mental illness (SMI) often struggle to manage their diabetes due to emotional and psychological difficulties, socioeconomic constraints, and fragmented care (27–31). Furthermore, Stenov et al.’s (32) interviews with patients living with SMI and diabetes showed that such patients experience a lack of support for their diabetes from mental healthcare services and that their mental health status is prioritized over their physical health, even in non-psychiatric healthcare settings. These trends have been confirmed by healthcare providers, who have expressed uncertainty about patients’ physical health, which can stem from a lack of knowledge and attitudes that prioritize psychiatric conditions (33–35).

Although qualitative studies have examined how people with SMI experience diabetes care, research focusing specifically on individuals with schizophrenia - particularly those with cognitive and functional impairment - remains limited (36–38). What remains largely unexplored is how more pronounced cognitive or functional difficulties affect the day-to-day management of diabetes in schizophrenia. Such challenges may considerably hinder self-care, making it essential to understand the lived experiences of those facing the greatest functional demands to inform the development of more effective and integrated care strategies. Therefore, this study explored the daily lives of individuals with both schizophrenia and diabetes, including those with cognitive and functional impairments, to better understand their health-related experiences and interactions with healthcare services.

## 2 Materials and methods

### 2.1 Study design and setting

Following a qualitative descriptive phenomenological approach, this study sought to explore the lived experiences of individuals living with both schizophrenia and diabetes. In line with descriptive phenomenology, the analysis was guided by the principles of openness, questioning pre-understanding, and adopting a reflective attitude to remain close to participants’ life world. Data were analyzed inductively using a thematic analysis grounded in descriptive phenomenology (39).This article adheres to the consolidated criteria for reporting qualitative research (COREQ) (40).

The study was conducted at four outpatient psychosis units at Sahlgrenska University Hospital. All units use case management, meaning that every patient enrolled has a clinical case manager responsible for coordinating care within the healthcare system (i.e., including annual health checks) and social services. Overall, the units serve approximately 1,650 patients (41). One of the outpatient units, hereafter referred to as the “complex outpatient unit” (COU), serves as a referral unit for the other units and specializes in patients with the most severe, complex functional impairments. These impairments often include pronounced cognitive deficits and limited ability to manage daily activities, typically associated with treatment-resistant schizophrenia and multiple psychiatric or somatic comorbidities. Based on prior data from these clinics, 27% of patients in the COU had diabetes compared with 13% in the other units; 17% vs. 13% had cardiovascular disease (e.g., coronary heart disease), and 8% vs. 3% had chronic obstructive pulmonary disease (42). Accordingly, participants recruited from the COU were considered to have significant cognitive and functional impairments based on the unit’s referral criteria and routine clinical assessments, rather than on a specific cognitive test.

### 2.2 Sample

Patients diagnosed with schizophrenia and either type 1 or type 2 diabetes were invited to participate by personnel at their outpatient unit following a consecutive sampling approach. For compensation, prospective participants were offered a gift certificate worth 100 Swedish kronor to a local grocery store. Patients whose mental health team judged that participation would place the patients at risk of harm were not invited, the assessment was based on the team’s clinical knowledge of the current severity of the patients’ psychotic symptoms. After conducting (around) 20 interviews, the data indicated signs of being saturated, as responses exhibited consistent patterns with no additional unique insights. Two further interviews were conducted to corroborate data saturation. Altogether, 22 participants were included in the study, 10 of whom were recruited from the COU (**Table 1**). Ethnicity was not recorded, all participants lived in the Gothenburg urban region and received care within the Swedish public healthcare system.

**Table 1:** Participants’ characteristics and length of interviews (N = 22).

Sample characteristics	N
Gender	
Female	7
Male	15
Age	
35–55 years	11
56–70 years	9
> 70 years	2
Years within psychiatric care	
5–10 years	3
10–20 years	6
20–30 years	7
30–40 years	5
> 40 years	1
Length of interview	
<10 min	2
10–20 min	9
20–30 min	6
30–40 min	2
>40 min	3

### 2.3 Data collection

Interviews were conducted by two mental health nurses and three health educators from the outpatient units in order to ensure that participants were familiar with the interviewers. The interviewers received preparatory training in qualitative interviewing and the use of the interview guide to ensure a consistent approach. However, the interviewers did not hold primary roles in the participants’ care (case manager or psychiatrist) to promote their sense of safety and willingness to participate. In one instance, a participant’s initial interview lasted only 6 min; the individual was offered a different interviewer and subsequently completed a follow-up interview that exceeded 50 min. Each interview was conducted in a location chosen by the participants (i.e., their outpatient unit or home). All interviews followed a semi-structured guide and were audio-recorded, transcribed, and stored according to the university’s research guidelines.

### 2.4 Data analysis

Data were subjected to thematic analysis grounded in descriptive phenomenology, which generally emphasizes being open, questioning preunderstandings, and adopting a reflective attitude to explore lived experiences (39). The research group read the transcripts, and the first author, MZN, listened to all interviews to familiarize herself with the material prior to coding. The transcripts were entered into NVivo version 12 (Lumivero). Initial coding was conducted by MZN and iteratively discussed with the main research group in order to incorporate diverse perspectives, ensure a thorough interpretation, and achieve consensus. Such a collaborative process helped to ensure the reliability and depth of the analysis. The research group consisted of both nurses, psychologist, and physicians with expertise in psychiatric care (i.e., MZN, CH, and PS) and diabetes care (i.e., CSL, KEO, and PS). Concept maps in NVivo ensured that themes and codes closely mirrored the empirical data. **Table 2** presents an example of the analytical process, including the development of subthemes, themes and their connection to the data. While the multidisciplinary composition of the team strengthened the interpretative depth of the analysis, we also recognized that our professional backgrounds could shape how we understood participants’ accounts. To maintain reflexivity, the research team continuously discussed potential preconceptions and critically reflected on how our roles and experiences might influence interpretation. These reflections were integrated into the coding and theme development process, helping to ensure that participants’ narratives remained at the core of the analysis.

**Table 2:** Example of thematic analysis process.

Quotations	Code	Subtheme	Theme
“It’s different trips, like traveling once or twice a year. That gives me something to look forward to. Otherwise, it’s probably my friends and relatives who motivate me to stay in good shape.”	Motivation to stay healthy	Finding motivation and meaning in health	Perceptions of health shaped by personal narratives
“I take a vitamin pill every day. It makes me feel a bit more energetic. I collect deposit cans every day. That way, I get some extra money, exercise, and movement.”	Strategies to facilitate healthy habits	Coping and support in daily life	Struggles of navigating health in daily life
“I’d like to have more follow-ups for my diabetes. To get appointment letters sent home. Right now, I’m just hoping everything’s okay, but last time it was too high, and that’s when I got this injection.”	Absence of interventions to support health	Seeking hands-on support in daily self-management	Need for formal support in managing health

### 2.5 Ethical considerations

All participants received written and verbal information about the voluntary nature of participating in the study, data confidentiality, and their right to withdraw at any time. For participants with severe cognitive deficits, verbal explanations were adapted until they confirmed their understanding and provided their consent. During interviews, two participants expressed reluctance to continue; those interviews were paused, and the participants were reminded of their right to withdraw at any time. Both participants ultimately chose to complete the interviews and remained in the study.

## 3 Results

**Figure 1:**
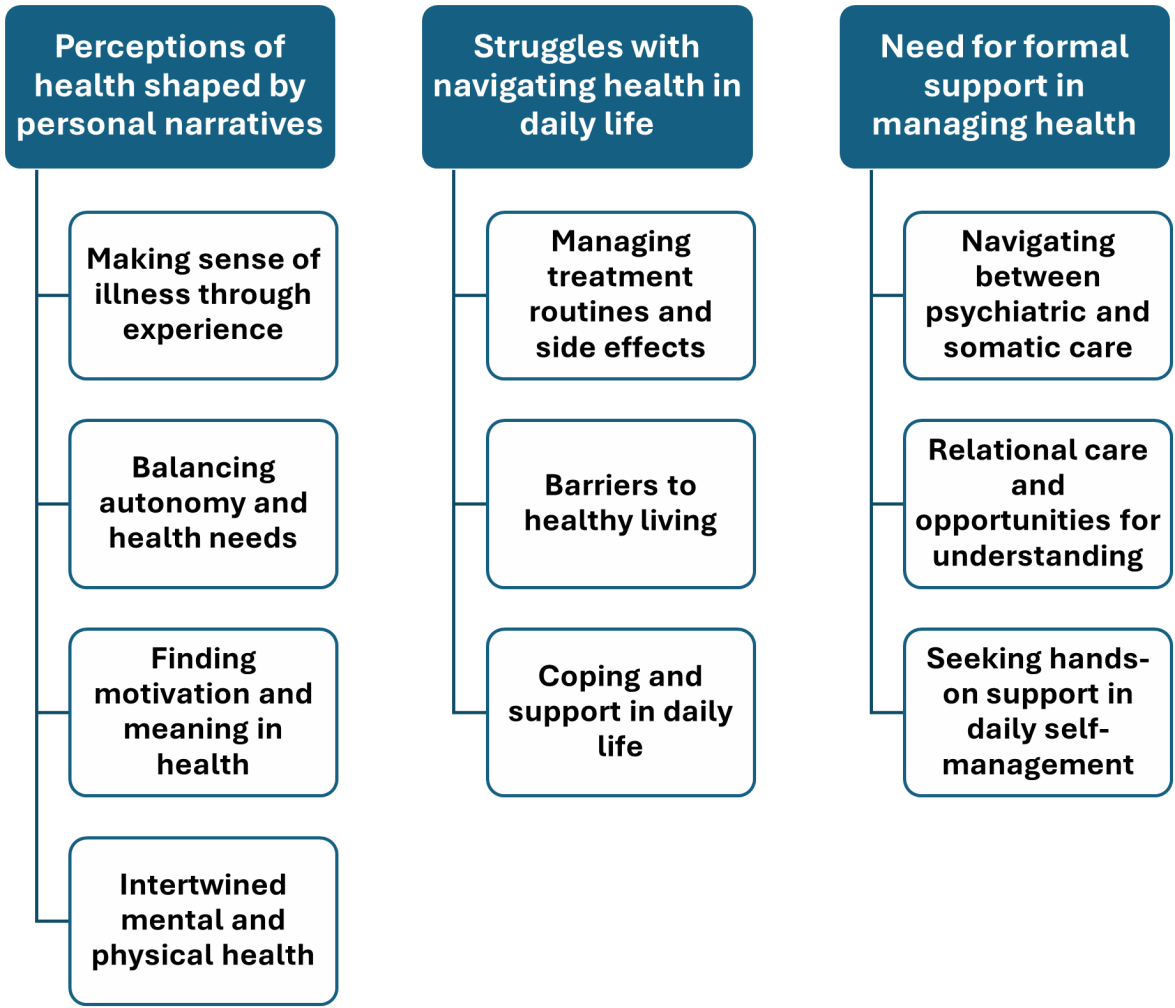
Overview of themes and subthemes.

Three themes regarding experiences with health-related aspects in everyday life while living with schizophrenia and diabetes emerged: (1) perceptions of health shaped by personal narratives, (2) struggles with navigating health in daily life, and (3) need for formal support in managing health. Each theme comprised several subthemes that further illustrate the nuances of participants’ experiences (**Figure 1**). The participants sometimes referred to the diseases jointly and sometimes separately, depending on how the illnesses affected their daily lives. Quotes are included to illustrate how the results are grounded in the participants’ accounts and to highlight their direct connection to the data.

### 3.1 Perceptions of health shaped by personal narratives

#### 3.1.1 Making sense of illness through experience

When asked about their current perceptions of their health, some participants first discussed their health history, and it became evident that past experiences significantly influenced their interpretations of their health at present. For some, the recognition involved acknowledging the ongoing impact of longstanding illnesses. For others, it reflected a narrative of persistent uncertainty since their initial diagnosis (e.g., psychiatric diagnosis), for they had struggled to comprehend why they had been diagnosed. Such ongoing uncertainty continued to shape their perceptions of their overall health. Understanding their illnesses and the management of them facilitated comprehension and an acceptance of their circumstances:

I used to have less understanding myself, but that’s because I didn’t know what it [schizophrenia] was about. But as soon as I gained insight, I especially began to understand care and treatment. Courses have been among the things that have helped me to gain that insight, and over time, I’ve come to understand what my diagnosis means. I’ve really appreciated that. Over the years, I’ve come to realize, and now I know, what it’s all about and why I need the medication. (Participant 1)

The manageability of illness was also influenced by the individual’s existential perspective. Whereas some changed their perspective as their illnesses progressed—for example, feeling threatened by their diabetes prompted a sense of guilt every time they consumed sugar—others remained calmer. Some expressed resignation or apathy regarding their illnesses, which could result in days spent bedridden. Others, however, chose to consciously ignore their illness and continue living their lives seemingly unaffected by diabetes:

I have diabetes, but I’m basically living as I always have. I still eat chocolate and things like that, and I don’t really think about having diabetes. I don’t want to obsess over it, so I just eat how I did before I was diagnosed. [ … ] I don’t want to think about being diabetic, but I’m not entirely sure if that might come back to haunt me later. (Participant 4)

#### 3.1.2 Balancing autonomy and health needs

For some individuals, autonomy in managing their care and treatment was more important than the actual need for care. Past experiences with coercive care and measures within psychiatric care, reinforced the importance of being in control of one’s care in order to achieve a sense of well-being, instead of simply seeking better health outcomes. For example, one participant reflected on knowing that if he didn´t feel well he could benefit from admission to psychiatric inpatient ward, but that he would never do it willingly. Routine physical examinations such as blood pressure and blood glucose monitoring could be perceived as being coercive if performed regularly without the patient’s initiative:

INTERVIEWER: Do you want any kind of examination at all?

RESPONDENT: No. I’d rather avoid it.

INTERVIEWER: But how are they supposed to know what medications you need if they don’t examine you? Because you don’t want them to examine you, right?

RESPONDENT: No, no.

INTERVIEWER: But how are they supposed to know?

RESPONDENT: I think it’s unnecessary to force care on someone who doesn’t want it.

INTERVIEWER: But how do you think they should figure out which medications you need? How should they know you need the ones you’re currently taking? Do you have any ideas?

RESPONDENT: They probably have computers with my info on them [ … ] like what medications I use and what care I consent to. I don’t want forced care. I’d rather stay at home and have a safe space where I can feel secure. (Participant 12)

Taken together, this indicates that earlier experiences of coercive psychiatric care shaped how some participants perceived routine health monitoring in both psychiatric and diabetes-related contexts.

#### 3.1.3 Finding motivation and meaning in health

Regarding motivations for maintaining health (e.g., making choices they considered to foster their health), the primary drivers were often relationships with friends and family, along with the desire to engage in valued activities such as traveling, writing, and collecting recyclables outdoors. For some participants, however, motivation was difficult to express; when asked what drove them to care for their health, they hesitated or gave vague answers such as “I don’t know.” These reluctant or uncertain responses may reflect a lack of motivation. Concerns about avoiding premature death from illness and recognizing improvement in well-being when practicing self-care served as strong motivators to continue healthy behaviors. Meanwhile, feelings of togetherness and a sense of belonging were considered to be crucial. Nevertheless, achieving such a sense of community could be difficult due to both psychiatric and physical illnesses, which made individuals feel different from the general population. Consequently, the opportunity to participate in group activities without fear of stigma was highlighted as an important factor in improving overall health:

I feel that small things, like hair loss, affect this. And it affects your confidence—you don’t want to stand out; you want to be like everyone else. The group sessions we had were good, because there you could see that there was a range—that some people had been ill longer, and just seeing that it’s possible to work and regain a sense of belonging. Maybe you don’t work, but you’re part of an association or have started a family. You’ve found some hope again, in a way. (Participant 7)

I think that it [health promotion activities outside of home] works best when I go through the counselor, because he knows me best. [ … ] We’ve decided in advance that we’ll meet, maybe go to a flea market or something and look around. [ … ] I guess I miss that a bit, yes, maybe meeting groups so that you can talk about being in the COU or something like that. (Participant 14)

#### 3.1.4 Intertwined mental and physical health

Participants acknowledged the connection between mental and physical health and the interplay between mental and physical states. Some observed that their physical condition influenced their mental state; for instance, poorly managed diabetes worsened fatigue, mood, and paranoid thinking. Conversely, others noted that their mental well-being had a greater impact on their physical health. For example, hearing voices interfered with their ability to attend to their physical needs, including maintaining an appropriate diet:

I think that my blood sugar is too low, and that makes me more paranoid—worse than usual. No, I need to get it together. I need to eat more food; I think I’m eating too little. I need better routines around meals. (Participant 9)

Another participant described a similar interplay, explaining how stress and emotional dysregulation influenced their eating habits and diabetes management

It feels like the better I feel, the better I can take care of my diabetes; it feels like it’s self-inflicted. I developed diabetes after eating very large amounts of food. I felt stressed and ate a lot. I should perhaps have told you [refers to the psychosis unit] more about this, for example. And the healthcare providers before you as well. I feel a great deal of stress, and the only thing that relieves it is food. (Participant 7)

Together, these statements could illustrate how the prioritization of mental and physical health may fluctuate depending on symptom intensity and perceived control over one’s physical condition. The prioritization of mental versus physical health varied among participants. One individual expressed relief upon experiencing increased physical illness after years of struggling with schizophrenia. They felt that their body had taken over and that their well-being was no longer so dependent on their mental state.

### 3.2 Struggles with navigating health in daily life

#### 3.2.1 Managing treatment routines and side effects

Experiences with health were often related to daily life, both in terms of barriers and facilitators. Independence in daily life, especially concerning medication and equipment for managing diabetes (e.g., blood glucose monitors) was seen as being pivotal, as was the ability to use the equipment independently. Access to medications at home and the ability to self-administer them were also crucial:

I’ve had diabetes for 10 years now. I treat it with both insulin and tablets. I manage to take my insulin on my own, I do. I handle everything myself. I’ve been given a lot of instructions over the years on how to take my insulin daily and how to manage it. (Participant 1)

Different medications, including ones for sleep deprivation, diabetes, various somatic issues, and antipsychotics, influenced participants’ daily health experiences. Side effects such as fatigue, impaired cognitive function, and weight gain were the most mentioned and were primarily attributed to medications for psychotic symptoms:

I hope that my medications don’t affect me too much, but I’m not sure. You always say the dose isn’t high, but I feel like I’m constantly struggling; my head feels stiff, and my thoughts aren’t the same. I mean, I used to be a completely different person, and now I have no drive. I’m just trying to make the best of the situation. (Participant 7)

Many also reported the positive effects of medication adherence that had eased daily life at home, especially after noticing that not taking medication worsened their situation, whether it was medication for anxiety, psychotic symptoms, or diabetes medication. Some reported tolerating negative side effects and remained adherent to their psychiatric medication because they had learned from experience that discontinuing antipsychotics led to worse consequences for their well-being. Medication, both for diabetes and psychiatric symptoms, was thus seen as being essential to maintaining health and as a potential barrier when side effects interfered with maintaining a healthy lifestyle. For example, monitoring blood glucose levels alongside medication clearly demonstrated the benefits and reinforced the commitment to remain adherent despite challenges.

#### 3.2.2 Barriers to healthy living

The participants additionally reflected on their lifestyle habits and how those habits impacted their health. Dietary habits, physical activities, smoking, and sleep patterns were also discussed. Nearly every participant had knowledge of what are considered to be healthy lifestyle habits and recognized that such habits could improve their health and that there was room for improvement. The participants from the COU, however, had a relatively difficult time imagining how such habits could be adopted. For example, one participant reported learning more about healthy lifestyles by reading books at the library. He explained how to obtain a library card but noted that he would need an identification card, which he lacked and therefore unable to use the library. Physical pain and financial constraints were other common barriers to participating in health-promoting activities that affected participants regardless of their level of functional impairment. However, the comorbidities of schizophrenia and diabetes appeared to amplify the impact of those barriers, for instance, among participants who knew that exercise was essential for managing their diabetes.

Given the barriers to healthy lifestyle habits in everyday life, a need for support to manage health emerged. In this context, “managing health” referred to assistance with basic day-to-day activities that were necessary for maintaining diabetes and overall well-being, including help with cooking or grocery shopping, picking up medication from the pharmacy, and changing batteries in blood glucose monitors:

I’ve been given test strips and stuff: the whole setup. There was this guy who was really nice and sold me cheap batteries; he said it was just because I was a member. They cost only 18 kronor. Anyway, I’ve bought new ones now. I even got help putting the batteries in at the store. But I also got help from the doctor at the health center. He wrote down exactly which batteries I needed to buy and gave me a note with the details, and then I went to the store and bought new ones. (Participant 9)

#### 3.2.3 Coping and support in daily life

When asked if they had anyone to support them with maintaining health in their everyday lives, participants gave different responses. Some mentioned that family and friends provided valuable support by assisting them with practical tasks such as giving advice and helping with cooking. Support could also manifest in the family’s willingness to learn about the individual’s illnesses in order to provide the best possible support. By contrast, others stated that they completely lacked support and were managing life as best they could on their own. For some of them, the choice seemed to be deliberate, while for others it appeared to be dictated by circumstances due to their illnesses and beyond their control:

A lot of my life is pretty lonely. I don’t really have much emotional contact with my family either. I don’t get much help, so I’ve had to stand on my own. I’ve lost a lot of friends, and I’m not sure that I have much support, really, if I look at it honestly. (Participant 7)

One participant said that managing their diabetes consumed so much energy in their daily life that it negatively impacted their relationship with friends and left them with no time or energy to engage in friendship. Another participant said that he had not told his family about his diagnosis with diabetes because he felt ashamed that he was not only mentally ill but now also physically ill.

Participants stated that they used their own strategies, including various distractions and self-education about symptoms, to make the symptoms more manageable. Education was used to understand and handle diabetes symptoms, including recognizing the sensations associated with low or high blood glucose levels, whereas distractions were primarily used to cope with psychotic symptoms:

I used to hear things like that [voice hallucinations] when I was about to eat. But now that I’m at home, I can listen to the radio while I’m eating, and then it’s not strange at all. I listen to the radio, I realize that I’m going to eat, and then it goes a little better. (Participant 9)

### 3.3 Need for formal support in managing health

#### 3.3.1 Navigating between psychiatric and somatic care

The third and final theme captures how formal healthcare systems and services impact individuals’ well-being. The formal support provided, encompassing psychiatric, somatic, and community-based care, significantly influences individuals’ health experiences. Most participants acknowledged that their psychiatric care includes health checks, primarily conducted through blood samples, and some also mentioned health discussions. Moreover, they acknowledged health assessments conducted by primary care physicians and dieticians for diabetes management. Many participants attending regular outpatient psychosis units reported receiving annual health checkups through primary care, whereas ones associated with the COU primarily recalled the psychosis unit conducting the physical health checks. However, some individuals in both groups did not recall undergoing any health checks whatsoever. Annual health checks involving blood samples could be perceived as uncomfortable but necessary, nonetheless. Participants with type 1 diabetes described diabetes care provider (e.g., specialist diabetes care) as more concrete because recommendations were directly connected to clear actions and visible results. For instance, they might be advised to adjust their diet and then meet with a dietician, followed by seeing the effects of these changes in their blood glucose readings. In contrast, psychiatric care was experienced as less direct, with treatment discussions often perceived as abstract or lacking immediate, practical steps.

#### 3.3.2 Relational care and opportunities for understanding

Regarding experiences with healthcare treatment, participants expressed a desire for minimal medication, whether for diabetes or psychiatric symptoms. When medication was required, the nurse’s or psychiatrist’s approach had a significant impact on the experience. The way that individuals were treated and approached by healthcare providers was consistently emphasized as contributing significantly to their perception and acceptance of their treatment. Overall, being treated with respect and being encouraged were highlighted as crucial factors in their willingness to engage in health interventions:

INTERVIEWER: What do you think could be done to motivate people in that regard [participate in health interventions]?

RESPONDENT: Well, instill a true, important belief that everyone can improve and develop their health and quality of life. It would be unfair if only certain people had the right to have the best health. Everyone essentially has the right to exist and grow, to improve their quality of life. (Participant 13)

When discussing what is lacking in health interventions from the healthcare system, whether psychiatric, medical, or community care, two main concerns emerged. The first was the desire for greater access to psychological therapy. In both groups, many participants reported not being offered psychotherapy to explore their personal health histories, particularly at the onset of their psychiatric illness, but emphasized that such therapy is essential for understanding themselves and ultimately achieving a sense of health:

Early on, I was supposed to talk to a psychologist, but that never happened. I would’ve wanted to go to therapy, but now I guess I’ve pretty much figured things out, most of it. Yeah, I’ve had to fight through it by myself. There are a lot of thoughts about my mental illness, about why it happened and stuff. I’ve never really gotten to talk about it. (Participant 7)

On the contrary, several participants who did not request psychotherapy stated that they had been given a lot of resources and time to better manage symptoms and navigate life, both in mental and physical aspects, which had given them a better understanding of themselves:

You have to be aware that there are many people in society who all have to share everyday life together, you know. It’s really important to be able to manage your illness, so nobody else gets hurt and so you don’t either. It’s really important to be aware of those things. I’ve had plenty of time to talk about it, and over the last 10 years I’ve gained a real understanding of the diagnosis. (Participant 1)

#### 3.3.3 Seeking hands-on support in daily self-management

Participants described needing practical, hands-on support to carry out everyday activities that were essential for managing both their diabetes and their mental health. This included help with physical activity, cooking groups, and preparing healthy meals, activities directly connected to diabetes self-management. It also involved support in leaving home or attending appointments, which helped stabilize their mental health and daily routines. Several participants explained that without someone to accompany them, they were unable to engage in activities they wished to do, including those necessary for maintaining their physical health. They often expressed a wish for healthcare staff to provide this kind of hands-on support, for example cooking together on a regular basis, learning how to prepare healthy meals, or shopping for groceries. Although such tasks do not necessarily require specialized healthcare expertise, participants explicitly sought this support from formal services.

Some had received this type of support from their psychosis units, such as being accompanied to the gym or on walks, but said they would stop those activities if the accompaniment were discontinued. For some participants, taking walks with their case manager was their primary way of engaging in physical activity, making the case manager’s presence essential for sustaining these health-related routines:

INTERVIEWER: What makes it difficult for you to manage your physical health? What is it that…?

RESPONDENT: If my case manager doesn’t show up.

INTERVIEWER: Ok, if your case manager doesn’t accompany you. But are there any other kinds of obstacles, physical or psychological [ … ]

RESPONDENT: No. No. No. (Participant 16)

This exchange illustrates how the absence of the case manager directly disrupted the participant’s ability to carry out a routine physical activity that supports diabetes management.

## 4 Discussion

### 4.1 Overview of key findings

Our findings reflect the unique health experiences of individuals living with both diabetes and schizophrenia, including complex functional impairments and often severe cognitive deficits. This study adds new insight by showing how individuals with significant psychiatric and cognitive challenges conceptualize health, interpret symptoms, and carry out daily self-management under cognitive, social, and systemic constraints. The findings suggest that personal narratives shape individuals’ perceptions of their health and that both healthcare and informal support can play a crucial role in managing health in everyday life. By highlighting the experiences of individuals who are typically excluded from research (37), this study offers a more detailed and nuanced understanding of self-care in the context of co-occurring schizophrenia and diabetes. It also highlights the crucial role of specialist professionals who provide consistent, concrete, and holistic support to sustain these efforts. Although participants with type 1 and type 2 diabetes differed in treatment routines and follow-up, their overall experiences of navigating health and care were largely similar. Both groups described challenges related to balancing mental and physical symptoms, implementing lifestyle changes, and relying on formal and informal support for daily management. However, participants with type 1 diabetes tended to describe their diabetes care, often provided by specialist diabetes services, as more concrete and understandable, particularly in relation to why specific measurements or routines were necessary.

### 4.2 The role of knowledge, education and support systems

Research has shown that individuals with psychotic disorders generally have relatively little knowledge regarding physical illnesses and health promotion (43, 44). Our findings indicate that people with schizophrenia and comorbid diabetes are aware of preventive lifestyle habits but struggle with executing them, as seen in participants with and without severe cognitive deficits. Those findings corroborate what Mulligan et al. (30) found: that suboptimal diabetes self-management among individuals with SMI is due not to their misunderstanding of the recommendations or consequences of poor glycemic control but rather a lack of support. In our study, the participants’ knowledge of healthy lifestyle habits may be attributed to their diagnosis with diabetes, which often involves receiving information and education about the illness and preventive measures.

Participants emphasized the importance of educational support from healthcare providers and how it enhanced their understanding of their overall health and treatment. By contrast, when such support was absent, they expressed a clear need for it. That observation corroborates previous reports indicating that people with SMI view their diabetes as a severe illness and that healthcare providers are not sufficiently engaged in their physical health (31, 32, 45). Support from family and healthcare providers was recognized as a key factor in facilitating effective diabetes self-management. As captured in the theme indicating the need for formal support in managing health, appreciated forms of support were often practical, seemingly simple yet critical forms of support, such as cooking assistance or help with accessing blood glucose monitors. These tasks, while seemingly basic, are essential to managing chronic illness. Without reliable support systems, even well-informed individuals may be unable to sustain health-promoting behaviors. The importance of support in daily self-management and the impact of healthcare relationships, resonate with findings from the general diabetes population (46).

### 4.3 Cognitive deficits and their impact on self-care

Our results also reveal unique challenges related to cognitive deficits, psychiatric symptoms, and stigma, underscoring the need for tailored approaches in this group. Participants described challenges in executing tasks such as changing the batteries in their glucose monitors, applying dietary advice consistently, and leaving home for exercise. These challenges underscore how cognitive impairments—such as difficulties with planning, memory, and initiating tasks—can directly hinder self-care behaviors and treatment adherence. These findings echo prior work showing that cognitive dysfunction in schizophrenia can hinder self-care behaviors and treatment adherence (20, 47). In addition to the well-documented loss of years of life (19), poorly managed diabetes also carries a substantial financial burden for both individuals and health systems, as complications such as cardiovascular disease, neuropathy, and hospitalizations increase healthcare costs and limit participation in work or daily activities (48). Addressing these challenges through coordinated and preventive care is therefore not only a clinical and ethical priority but also an economic one.

### 4.4 Self-understanding and recovery-oriented perspectives

Self-understanding was emphasized as being crucial for both experiencing good health and maintaining over time. Many participants’ primary desire for healthcare support to better manage their health was psychotherapy so that they could understand their own history of illness, especially their psychiatric illness. The definition of health should be understood as encompassing both objective medical assessments and subjective experiences shaped by the individual’s values and personal narrative (49). The subjective understanding of one’s health is also identified as a key driver of health behaviors such as seeking care, adhering to treatment, and making healthy lifestyle choices (50). Such understandings are particularly important among individuals with chronic diseases in order to maintain a coherent sense of self. This is especially relevant for individuals with schizophrenia, since their narrative identity, that is, the internalized and evolving life story through which they make sense of illness and self, may be disrupted and often presents with a disjointed structure (51). The emphasis on personal narratives and self-understanding resonates with recovery-oriented models of mental health care, such as the CHIME (Connectedness, Hope, Identity, Meaning, and Empowerment) framework (52). Integrating such recovery principles with holistic and multidisciplinary approaches could foster both psychological growth and physical health for individuals with complex comorbidities, shown to increase patient involvement, improve glycemic control, and reduce emergency department visits (53). Moreover, our findings advocate systemic accountability in managing health among those with dual diagnoses by providing coordinated care that addresses both practical needs and subjective well-being.

### 4.5 Future research and clinical recommendations

This study contributes new knowledge about how individuals with a dual diagnosis of diabetes and schizophrenia experience and manage health in daily life, emphasizing the value of integrated psychological, social, and practical support. Future research should explore structured interventions that enhance narrative identity, improve healthcare provider communication skills, and bridge gaps in service delivery for individuals with complex mental and physical health needs. When developing and implementing such interventions for people with significant cognitive impairments, it is essential to adapt information and support to their cognitive capacities by using clear, concrete, and repeated communication and by ensuring continuity of contact with trusted professionals. Such adaptations are especially important for sustaining daily self-management routines related to diet, medication, glucose monitoring, and physical activity. Interventions should incorporate hands-on elements such as guided practice in daily activities and collaborative goal setting to support learning and retention, as participants often required practical support to carry out the everyday tasks necessary to maintain stable self-management, as managing both schizophrenia and diabetes simultaneously increased the demands of these tasks. Additionally, approaches that integrate cognitive rehabilitation or compensatory strategies within psychosocial and physical health programs may further strengthen engagement and outcomes. Including participants with cognitive impairments in such work remains crucial.

### 4.6 Methodological considerations

Triangulating interviewers in our study was considered to be essential for enhancing the credibility of the findings by incorporating different perspectives and providing richer data. Because variations in the consistency of interviewers can impact the reliability of results, all interviewers were trained prior to data collection in order to obtain a shared understanding of the objective and how to use the interview guide.

Although interview length varied, approximately half lasted up to 20 minutes, the shorter interviews still provided information-rich descriptions of lived experiences. The trained interviewers used clarifying and follow-up questions to encourage elaboration and ensure depth despite the briefer duration. In one instance, a participant’s initial interview lasted only six minutes. The interview was repeated with a different interviewer, which resulted in a 50-minute interview that yielded considerably richer data. This experience highlighted the importance of interviewer–participant rapport and demonstrated how responsiveness and flexibility during data collection contributed to obtaining detailed and meaningful accounts.

Ten of the 22 participants were recruited from an outpatient unit specializing in psychotic disorders and severe functional impairments, often including pronounced cognitive deficits, a population whose experience is often absent in research (36, 37). The exclusion of some eligible participants based on mental health assessments introduces concerns about transferability, for it may limit the broader applicability of the findings. Although individual assessments were conducted, vulnerability can also be overestimated (54). However, we prioritized respecting the assessments of the mental health team, who likely had a better understanding of the participants’ well-being than the research group did.

## 5 Conclusion

Our findings highlight the unique health experiences of individuals living with both schizophrenia and diabetes, particularly those with additional cognitive impairments. Although participants understood the importance of preventive lifestyle behaviors, they often struggled to integrate and sustain them in daily life. Interventions that help individuals make sense of their illness history and foster a coherent sense of identity are crucial, as such subjective understanding influences health behaviors, treatment adherence, and meaningful lifestyle change.

From a clinical perspective, these results underscore the importance of coordinated care that addresses both cognitive impairments, physical health management and ensures comprehensive care that enhances treatment adherence and self-care. Healthcare providers play a key role in tailoring support to each patient’s lived experience, combining practical assistance with recognition of subjective well-being. Identifying personal resources, addressing barriers such as stigma and financial constraints, and providing integrated multidisciplinary care can improve outcomes and quality of life for this population. Ultimately, systemic accountability is needed to ensure that people with dual diagnoses receive comprehensive, holistic care that bridges psychiatric and somatic health.

## Data Availability

The raw data supporting the conclusions of this article will be made available by the authors, without undue reservation.
